# Genetic markers identify duplicates in Nordic potato collections

**DOI:** 10.3389/fpls.2024.1405314

**Published:** 2024-08-26

**Authors:** Pawel Chrominski, Ulrika Carlson-Nilsson, Anna Palmé, Hanne Grethe Kirk, Åsmund Asdal, Lena Ansebo

**Affiliations:** ^1^ NordGen Plants, Nordic Genetic Resource Center (NordGen), Alnarp, Sweden; ^2^ Danish Potato Breeding Foundation (LKF-Vandel), Vandel, Denmark; ^3^ Norwegian Genetic Resource Centre (NGS), Norwegian Institute of Bioeconomy Research (NIBIO), Ås, Norway; ^4^ Fredriksdal Museums and Gardens, Helsingborg Museum, Helsingborg, Sweden

**Keywords:** potato, SSR, microsatellites, genotyping, genebank, duplicates, genetic resources

## Abstract

**Introduction:**

The first small scale cultivation of potatoes in the Nordic countries began roughly 300 years ago, and later became an important staple food in the region. Organized conservation efforts began in the 1980s, and today, potato landraces, improved varieties, and breeding lines are conserved in genebanks at the Nordic Genetic Resource Center (NordGen), Sweden, and the Norwegian Genetic Resource Centre (NGS), Norway, as well as at potato breeding companies across Nordic countries. All these collections house a diverse array of genotypes with local names and local growing histories from the whole region. However, the presence of duplicates, and inconsistent naming has led to confusion.

**Methods:**

In this study, 198 accessions of cultivated potato (*Solanum tuberosum* L.) have been genotyped with 62 microsatellite (SSR) markers. The analyzed accessions came from three collections: 43 accessions from the Danish Potato Breeding Foundation in Vandel (LKF-Vandel), 90 from NordGen and 65 from NGS.

**Results and discussion:**

The genetic analysis revealed 140 unique potato genotypes and 31 groups/clusters of duplicates, most of which contained duplicate pairs and the others three to ten accessions. Several accessions with distinct names were genetically identical or very similar, suggesting historical sharing, and regional distribution of seed potatoes, leading to the emergence of diverse local names. Moreover, many improved varieties from early potato breeding were revealed to have duplicates that have been considered Nordic landraces. Furthermore, potato accessions with identical names but originating from different collections were confirmed to be duplicates. These findings have already influenced management decisions and will further improve management practices for Nordic potato collections. Additionally, this new knowledge will benefit Nordic potato breeding efforts and allow for the dissemination of more accurate information to other users of potato diversity.

## Introduction

The cultivated potato (*Solanum tuberosum* L.) has its origin in the Andes in South America and was domesticated about 7,000–10,000 years ago (Hawkes, 1990). It was brought to Europe in the second half of the sixteenth century and the first introductions were to Spain, via Canary Islands, and to England (Hawkes and Francisco-Ortega, 1992; 1993). It was however not until the nineteenth century that the potato became a staple of everyday diet for the rapidly increasing European population, and it ended the famine that especially northern Europe was struggling with (McNeill, 1999). The potato landraces related to *S. tuberosum* Chilotanum Group were particularly favored in northern Europe because of their good adaptation to a short growing season with tuberization under long daylengths. The improved varieties grown today are usually related to this group of Chilean potatoes (Spooner et al., 2005; Ríos et al., 2007; Ames and Spooner, 2008).

The potato reached the Nordic countries (Denmark, Finland, Iceland, Norway, and Sweden) later than the rest of Europe. The first records come from Denmark in 1630 (Tolstrup, 2001) and from Sweden in 1658 (Swederus, 1877), but at that time potato was more of a curiosity grown in botanical gardens. The history of growing potatoes for consumption dates back to the 1720s. In 1719–1720, French Huguenots settled in Denmark and started growing potatoes in the town of Fredericia (Kyrre, 1938). In Sweden, Jonas Alströmer brought potatoes from a trip to Western Europe and started growing them on his farm near Alingsås in 1724 (Alströmer, 1777). The potatoes that arrived in Denmark and Sweden originated in France, the Netherlands, Germany, and England, and they were then spread further across the Nordic region (Osvald, 1965; Erjefält, 2001). Until the mid-nineteenth century, these imported potatoes were often propagated by means of botanical seeds which resulted in many new genotypes (Osvald, 1965; Umaerus, 1989). Some of the still existing landraces may potentially have their origin in these efforts (Roer, 1992; Erjefält, 2001).

In the second half of the nineteenth century, commercial improved varieties with better resistance and agronomical traits originating in other European countries and USA, were imported to the Nordic region and became very popular (Osvald, 1965). Potato breeding in the Nordic countries started in Sweden at the beginning of the twentieth century and the first Swedish commercial variety was released in 1921. In the 1930s, successively Finland, Norway and Denmark released their first varieties (Bjor, 2001; Erjefält, 2001; Tolstrup, 2001; Varis, 2001). By the end of the twentieth century Nordic breeding institutions had bred 99 potato varieties in total (Veteläinen, 2001).

With the emergence of the improved potato varieties, the landraces were gradually withdrawn from cultivation. Nevertheless, an inventory of potatoes grown in Sweden in the 1940s identified 118 local and foreign genotypes. Among them a few 200 years old landraces were still present (Hagberth, 1951). According to later estimates (Veteläinen et al., 2005), about 70 potato landraces from all Nordic countries were conserved in genebanks. However, it is suspected that some of the landraces had already been lost by then.

With the development of plant breeding in Europe in the twentieth century, plant genetic resources came into the spotlight. Also, the Nordic countries recognized the need for conservation of genetic resources to prevent their loss and facilitate access. The Nordic Gene Bank (NGB) was founded in 1979 as a joint Nordic institution and became a part of the Nordic Genetic Resource Center (NordGen) in 2008 (Yndgaard and Solberg, 2019). Systematic work on the conservation of potato genetic resources began at NGB in the same year as it was established. The *in vitro* collection of potatoes at NordGen is continuously growing and currently contains 95 accessions, of which 40 are landraces, 46 improved varieties, and 9 breeding lines. The potato collection at NordGen is today considered to include most of the remaining Nordic potato landraces. The focus for acquisition has therefore shifted to collection of older improved potato varieties of Nordic origin and relevance.

Registration and certification of new cultivars revealed an issue of numerous synonyms among potatoes bred in the early twentieth century. In England in 1919, the National Institute of Agricultural Botany (NIAB) was established in Cambridge, and shortly thereafter, the Potato Synonym Committee was formed within its structure. Chaired by Redcliffe Salaman, the committee played a pivotal role in addressing this challenge, accurately describing potato varieties and eliminating the prevalent practice of marketing old and unreliable cultivars under new names (Salaman, 1926).

Potatoes grown in the Nordic countries have been morphologically described in different studies spanning over a hundred years (Øverby, 1929; Hellbo and Esbo, 1942; Hansen and Nissen, 1945; Hagberth, 1951; Veteläinen, 2001). Even today, the assessment of morphological characters is used to identify different potato genotypes, which is one of the basic tasks of genebanks conserving potato genetic resources. To build the Nordic collection, the morphology of more than 600 potato accessions were studied. Many of them did not meet the criteria for conservation at NordGen as they turned out to be non-Nordic genotypes or duplicates of accessions already present in the collection. In most cases, morphological characterization is sufficient to distinguish between different potato genotypes. However, in the case of genotypes that are closely related or infected with viruses, identification based on such an assessment can be difficult (Veteläinen et al., 2005). The morphological characters can also be influenced by environmental factors and, moreover, this method of potato identification is laborious and expensive.

With the development of molecular genotyping techniques, NordGen began to use these methods to identify potato accessions and study the genetic relationship between them. Veteläinen et al. (2005) studied the diversity of 32 Nordic potato landraces by means of amplified fragment length polymorphism (AFLP) markers and morphological traits. In a European project (Hoekstra and Reid, 2014), over four hundred potato accessions from seven collections were genotyped using 12 microsatellite (SSR) markers. Thirty-four accessions from NordGen were included in this study. Old potato clones with questionable identity were fingerprinted to confirm or correct cultivars’ names. In a more recent project Selga et al. (2022) studied diversity and population structure of Nordic potato landraces, improved varieties, and breeding clones.

Although NordGen has a mandate from all Nordic countries to conserve potato genetic resources, Nordic potato accessions are also included in other collections, for example the breeding company LKF Vandel in Denmark (now Danespo) and the Norwegian Genetic Resource Center (NGS). Both the NordGen and NGS collections contained a large number of potato genotypes that required identification. In our joint Nordic research project reported herein, we genotyped potato accessions from the three Nordic collections using 62 microsatellite markers (SSR). The main purpose of this study is to confirm the identity of suspected duplicates and identify any additional duplicates in the collections. This information has already been used to remove duplicates when relevant, and to ensure correct naming of the accessions.

## Materials and methods

### Study material

The potato germplasm used in this study consisted of 198 accessions of cultivated potato *Solanum tuberosum* L. Information regarding the studied accessions, such as name, accession number, collection in which a given accession is conserved, country of origin, clonal type, pedigree, and release date, are included in the **Supplementary Table S1**. The analyzed accessions came from three collections: 43 accessions from the Danish Potato Breeding Foundation in Vandel (LKF-Vandel) in Denmark; 90 from the Nordic Genetic Resource Center (NordGen) in Sweden; and 65 from Norwegian Genetic Resource Center (NGS), a part of the Norwegian Institute of Bioeconomy Research (NIBIO) in Norway. NordGen is a genebank shared by all Nordic countries and conserves potato genetic resources for the entire region. However, due to stricter phytosanitary regulations governing potato import and export to non-EU countries like Norway, NGS established its own potato collection for domestic use in cooperation with NIBIO Division of Biotechnology and Plant Health. The collection at LKF-Vandel maintains old varieties, and newer parental lines which are considered valuable for future breeding and cultivation of potatoes in Denmark. Although all three potato collections focus on genotypes originating from the Nordic region, foreign accessions may also be accepted if they have been grown on a wider scale in one or more of the Nordic countries or may constitute a valuable resource for potato breeding in the Nordic region. Of the potato accessions used in the study, 165 were of Nordic origin, while 33 accessions were from countries outside this region. The studied accessions contained genotypes of three different types: landraces, improved varieties, and breeding lines. The history of several landraces such as Raudar Islenskar (Gammal Svensk Röd), Jämtlands Vit and Leksands Vit used in this study dates to the 1720s. The oldest improved variety analyzed here was Garnet Chili from 1857 and the newest one was Superb from 2003. Several breeding lines with important traits and potential value for future potato breeding were also included in this study. In total, 49 accessions from NordGen and NGS were recently added into the collections, most with documentation suggesting that they could be landraces or old improved varieties. The potato accessions used in this study were conserved in genebanks in *in vitro* or field collections. Accessions accepted for long-term conservation are usually kept in *in vitro* collections.

### Genotyping

DNA was extracted from fresh or freeze-dried leaf tissue from one individual in each accession. The material originating from NordGen’s collection was extracted using the protocol described by Doyle and Doyle (1987) and modified as described in Hagenblad et al. (2023). Thereafter the concentration of DNA was measured using a spectrometer (Eppendorf) resulting in concentrations in the range 0,005–0,04 µg/µl.

The samples were genotyped in 2013–2015 with 62 microsatellite markers from a number of different sources (Kawchuk et al., 1996; Provan et al., 1996; Milbourne et al., 1998; Bradshaw et al., 2006; Ghislain et al., 2004; Feingold et al., 2005; Kørup Sørensen et al., 2007; Reid and Kerr, 2007; Szajko et al., 2008; Ghislain et al., 2009), see **Supplementary Table S2**. The markers were selected in order to have a good coverage of the genome and high power to distinguish among different accessions. To achieve this some 5–6 markers were selected in each chromosome and when there were good alternatives, the most polymorphic marker was selected. The markers have been used at LKF-Vandel to evaluate genetic relationships and potential degree of inbreeding in new crosses for plant breeding purposes.

Each marker was amplified separately by PCR in 96 plates with two sets of 45 accessions, 2 control samples and one blank. Fragment analysis was conducted on an ABI Prism 310 Genetic Analyzer (Applied Biosystems). The microsatellites were combined into 13 sets, within which the different markers were either separated by size or color. Allele sizes were analyzed with the GeneMapper software.

### Data analysis

Each amplified PCR product was scored as either present or absent (1 or 0) or if it was not possible to interpret, as missing data. This information was combined into a matrix including data on all fragments amplified at the different microsatellite loci, for all analyzed accessions (raw data available at https://doi.org/10.6084/m9.figshare.26325037.v1). This approach has frequently been used in potato (e.g. Muhinyuza et al., 2015; Duan et al., 2019; Spanoghe et al., 2022) to avoid problems with dosage determination in this generally tetraploid crop. The dataset includes 198 accessions analyzed with 62 microsatellite markers, resulting in 493 different amplified fragments, of these 208 are polymorphic in this data set. For each microsatellite marker, the total number of observed alleles and their size range was noted (**Supplementary Table S2**).

To identify duplicates, the number of pairwise differences among all accessions were evaluated with the program MEGA6 (Tamura et al., 2013). All allele positions with missing data were removed for each pairwise comparison. To get a general estimate of the amount of divergence between accessions, the number of pairwise differences was divided by the total number of bands (493). Trees were constructed with the program MEGA11 (Tamura et al., 2021) based on the number of differences between accessions. Methods explored were the UPGMA as well as the neighbor joining (NJ) approach and bootstrap with 500 replicates was used to estimate the support in the data of each branch. Also here, missing data was removed in all pairwise comparisons.

## Results

### Identification of duplicates/clones

**Figure 1:**
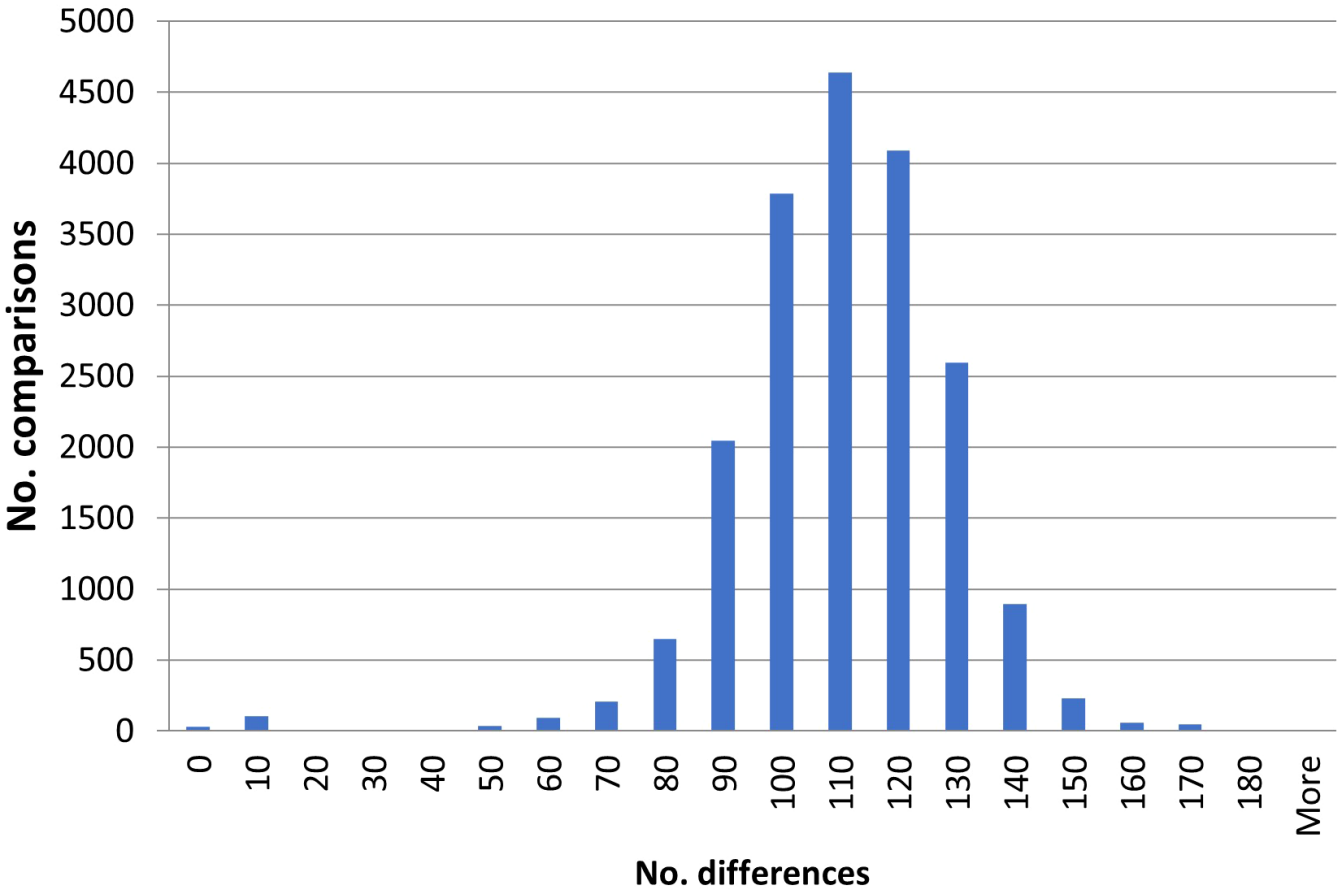
A histogram of the number of differences observed in pairwise comparisons of all analyzed accessions.

**Figure 2:**
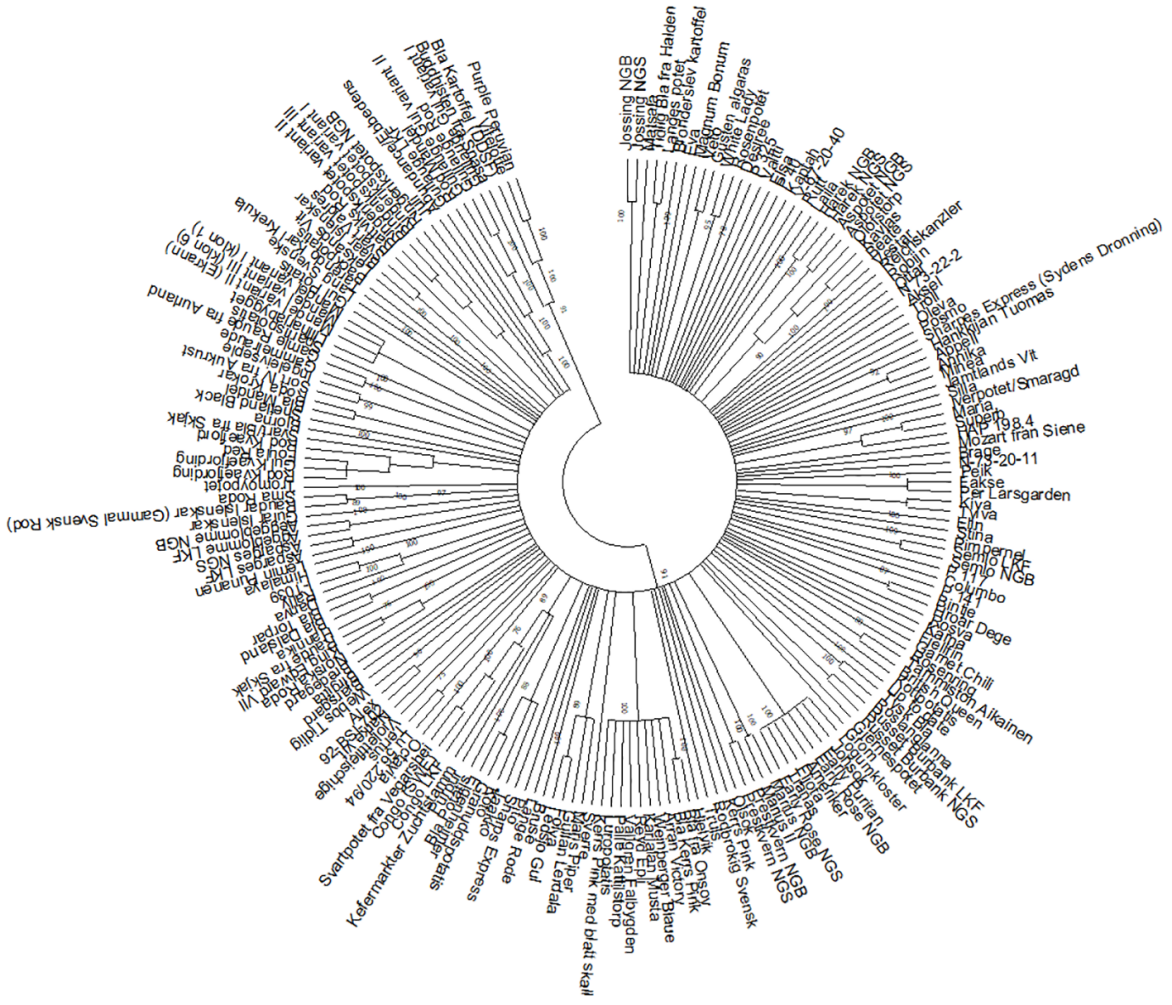
Dendrogram constructed with the UPGMA method with branches with less than 75% bootstrap support collapsed. All clusters described in **Table 1** are found in clearly delineated and well supported groups in this tree.

The goal of this study was to identify duplicates among the investigated potato accessions. This was done by pairwise comparisons among all the accessions. In total there are 32 pairwise comparisons with no differences, 34 with one difference (representing a difference in about 0.2% of the amplified bands and a similarity of 99.8%), 29 with two differences (0.4%), 26 with three (0.6%), 11 with four (0.8%) and two with five (1%) (**Supplementary Table S3**). There is a low number of pairs with between five and 40 differences. Most comparisons show between 70–140 differences (**Figure 1**) and the average is 106 differences. Based on this distribution, and the in general clonal propagation of potato, accessions with five or fewer differences are interpreted as duplicates or very closely related accessions originating from the same clone in the recent past. In the text these will be referred to as duplicates. Using this definition, there are 31 groups of duplicates/clonal clusters (**Table 1**). Of these, 21 are duplicate pairs, 4 contain three accessions and 3 four accessions and 1 five accessions. The remaining two are larger groups with eight and ten accessions respectively. The groups are also identified and well supported in the dendrograms (**Figure 2**; **Supplementary Figure S1**). In total, there are 89 accessions included in the 31 duplicate groups and the remaining 109 accessions had no duplicates among the investigated Nordic collections. This means that we have identified 140 unique genotypes.

**Table 1:** Groups/clusters of accessions that according to the investigated genetic markers are identical, or nearly identical.

Cluster	Accessions	No. accessions	No. observed differences
C-1	Arran Victory, Blå fra Onsøy, Blå Kerrs Pink, Karjalan Musta, Kerrs Pink med blått skall, Luröpotatis, Pålle Kättilstorp, Reyð Epli, Vallgren Falbygden, Weinberger Blaue	10	0 - 3
C-2	Ameriker, Early Puritan, Early Rose NGB, Early Rose NGS, Fljota, Jonsok, Løgumkloster, Tranås	8	0 - 4
C-3	Gullauge Gul variant I, Gullauge Gul variant II, Gullauge Rød, Gullauge LKF, Röd Mandel	5	0 - 2
C-4	Foula Red, Gul Kvæfjording, Rød Kvæfjord, Rød Kvæfjording	4	0 - 4
C-5	Lillhärjåbygget, Mandel variant I (klon 1), Mandel variant II (Ekrann), Mandel variant III (klon 6)	4	1 - 5
C-6	Ringerikspotet variant I, Ringerikspotet variant II, Ringerikspotet variant III, Ringerikspotet NGB	4	0 - 1
C-7	Beate, Kavlås, Olofstorp	3	0 - 2
C-8	Blå Kartoffel (DDSF), Purple Peruvian, Vitelotte	3	0 - 1
C-9	Congo (SWE), Congo LKF, Svartpotet fra Vegårshei	3	0
C-10	King Edward VII, Norska Röda, Raude fra Skjåk	3	2 - 4
C-11	Æggeblomme LKF, Æggeblomme NGB	2	0
C-12	Asparges NGS, Asparges LKF	2	0
C-13	Åspotet NGB, Åspotet NGS	2	3
C-14	Blå Dalsland, Blå Torpar	2	2
C-15	Blaar Islenskar, Svart Valdres	2	0
C-16	Eigenheimer, Granuddspotatis	2	0
C-17	Fakse, Per Larsgården	2	1
C-18	Folva, Ledsjö Gul	2	0
C-19	Gamle Raude fra Aurland, Gammelraude	2	0
C-20	Hårek NGB, Hårek NGS	2	0
C-21	Himalaya, I-1039	2	0
C-22	Jøssing NGB, Jøssing NGS	2	1
C-23	Kiva, Tylva	2	5
C-24	Lange’s potet, Tidlig Blå fra Halden	2	2
C-25	Maria, Superb	2	0
C-26	Marius II, Marius NGB	2	1
C-27	Prestkvern NGB, Prestkvern NGS	2	0
C-28	Raudar Islenskar (Gammal Svensk Röd), Små Röda	2	1
C-29	Roslanglänna, Tysk Blå	2	1
C-30	Russet Burbank NGS, Russet Burbank LKF	2	0
C-31	Semlo NGB, Semlo LKF	2	3

Each cluster includes accessions that are separated with five or fewer differences (representing a difference of 0 - 1%) and can be regarded as duplicates or very close relatives originating from the same clone.

### Genetic structure

The main aim of this study was not to investigate the overall genetic structure in the Nordic potato collections but to identify duplicates. However, the dendrograms (**Figure 2**; **Supplementary Figure S1**) clearly show some additional clustering of accessions, in addition to the duplicates. In some cases, two accessions group together, such as Veto and Gusten Älgarås, Appell and Annika, Bintje and Hroar Dege, Lü 56.220/94 and Tertus, Alex and 92-BSI-702, Röda Krokar and Blå Mandel, Sort IV fra Aukrust and Ingeleivseple. In other cases, there are larger groups involving duplicates as well as other accessions, such as Maris Piper and Gullan Lerdala clustering with C-18, Jaakko and Koto grouping with C-16, Blå potatis (Blå Märta), Purpur and Kefermarkter Zuchtstamm with C-9 and Svart/Blå fra Skjåk with C-4.

## Discussion

### Many unique genotypes in the Nordic collections

This study clearly identifies 140 unique genotypes that are currently conserved in the Nordic potato collections, including landraces, cultivars from different time periods (release years 1857 - 2003), and a few breeding lines (**Supplementary Table S1**). The majority of the accessions studied have their origin in one of the Nordic countries or have been cultivated in the region. Together, they represent a substantial amount of variation of relevance for the Nordic region and are important resources for future plant breeding efforts, research projects and, especially for the landraces, a part of our cultural heritage.

The majority of the modern varieties analyzed in this study were genetically unique and had no synonyms. Many of these unique accessions have been identified in previous studies. Veteläinen et al. (2005) confirmed, using AFLP markers, that the 32 potato landraces present in NordGen’s collection at that time were unique. Similarly, Selga et al. (2022) analyzed the accepted potato accessions at NordGen with SNP (Single Nucleotide Polymorphism) markers and confirmed the uniqueness of 73 of the 75 accessions that were present in the collection at that time.

### Duplicates with identical names

As potato is a clonally propagated crop, the risk of genetic changes after regeneration should be minimal compared to seed propagated crops. Having this in mind, accessions with the same name should be identical, or very similar, at different genebanks even if they are collected at different locations or have gone through several generations of regeneration. Nine of the observed clusters contained two accessions with identical names conserved at different genebanks (C-11, C-12, C-13, C-20, C-22, C-26, C-27, C-30, C-31) (**Table 1**). This result gives strong support to the hypothesis that both genebanks conserve the correct accession in these cases. Only in one situation, two accessions with the same name ended up as genetically different. Sharpes Express from the LKF collection differed from the accession with nearly the same name in the Norwegian collection. This indicates that one of the two collections likely preserves an incorrect accession under the name Sharpes/Sharps Express.

### Duplicates with similar names

Sometimes a genebank collection for different, often historical, reasons contain several accessions of the same landrace or improved variety. As every accession is an economic cost for the genebank, it is wise to carefully consider if such duplicates should be kept or not.

In our study we found three clusters, C-3, C-5, and C-6 including duplicate accessions of the landraces/improved varieties Gullauge, Mandel and Ringerikspotet respectively (**Table 1**). The biggest cluster (C-3) includes, besides Gullauge LKF, three different clones named Gullauge; Gullauge Gul variant I, Gullauge Gul variant II and Gullauge Rød. These three clones are all conserved at NGS in Norway. The results both confirms that the clone from LKF, Denmark, is identical or genetically very close to the two yellow (gul) variants conserved at NGS but also that the two Norwegian yellow variants themselves are genetically very similar, and it might be recommended to only keep one of them in the collection. The origin of Gullauge is unknown but it has a cultivation history in Finland, Iceland, Norway, and Sweden. It can therefore be suspected that Gullauge LKF originally came from Sweden or Norway.

In addition, the morphologically divergent red skinned clone Gullauge Rød cannot be genetically separated from the three yellow skinned ones. This indicates that Gullauge Rød is a skin color mutant with the same origin as the yellow skinned Gullague, which is found both at LKF and NGS, and consistent with what has been shown for other skin color mutants (Kishine et al., 2017). This gene is probably not situated within any of the fragments amplified by the 62 microsatellite markers in this study. We observed the same result in cluster C-4 where two accessions of Kvæfjording, namely Rød Kvæfjording and Gul Kvæfjording (both from NGS), did not differ genetically according to our results, even if they have different skin color.

The fifth accession in cluster C-3 is Röd Mandel, a red skinned clone conserved at NordGen. According to the documentation, this accession came to Sweden (Jämtland, Härjedalen county) from Trondheim, Norway, maybe around 1866–67. At NordGen this accession has been conserved under the name Röd Mandel, maybe because of morphological similarities with the variety Mandelpotatis. However, the result of our study confirms that the correct variety is Rød Gullauge. As ordinary Gullauge (with yellow skin) is already conserved at NordGen, this finding resulted in the rejection of the accession Röd Mandel/Rød Gullauge.

The second cluster, C-5, includes three clones of Mandelpotatis (Mandel potato/Almond potato). This name is a collective designation for a certain type of potato varieties. The tubers have quite a typical morphology, small and very elongated, slightly flattened, and often somewhat crooked. The skin is thin and smooth, eyes shallow, and the flesh yellow. In addition to the regular Mandel, with white flowers and white skin, accessions with violet flowers and white skin and white flowers and red skin respectively are also available (Hellbo and Esbo, 1942). The variety name Mandelpotatis can be found already in variety lists from the beginning of the 19^th^ century (Osvald, 1965) but in different countries as well as districts this type of potatoes can also be found under other names, often deriving from persons, locations, provinces etc (Hellbo and Esbo, 1942). In Sweden, this old landrace has traditionally been cultivated mainly in the northernmost provinces. In Norway it is called Mandelpotet, Langpotet or Krokeple and it is cultivated in northern Østerdalen and Gudbrandsdalen as a special quality potato. Mandelpotet from Oppdal is sold under a protected geographical indication: Fjellmandel fra Oppdal (NIBIO, 2020b). The variety is also grown in Finland under the name Lapin puikula or Puikula.

In our study, five accessions with the name Mandel were included. Out of them, Röd Mandel was found in cluster C-3 (described above) and is most certainly a misclassification. Three were included in cluster C-5 (Mandel variant I (klon 1), Mandel variant II (Ekrann), Mandel variant III (klon 6)), all from NGS, and one (Blå Mandel) showed to be a unique accession not genetically close to any other accession in the study.

The results show that the three Mandel variants from the NGS collection are duplicates and it could be considered if they all need to be kept in the collection. Blå Mandel is most certainly an old landrace, grown in the Swedish provinces Västerbotten, Norrbotten and Lappland. It is said to be the most cultivated variety in Sweden before the white skinned Mandel was introduced. According to the results in our study, Blå Mandel is genetically different from the three Mandel accessions in the C-5 cluster and instead related to the accession Röda Krokar (mainly cultivated in southern Norrland) (**Figure 2**; **Supplementary Figure S1**). Hellbo and Esbo (1942) consider Röda Krokar as a synonym to the white flowering, red skinned Mandel and the synonym name Röd Mandel is also given in Veteläinen (2001). Even if the color of the skin is different between Blå Mandel (dark blue skin) and Röda Krokar/Röd Mandel, they both have white flowers, and our results show that they are genetically close to each other. It is however somewhat unexpected that these two Mandel varieties do not cluster with the accessions in C-5 containing the other Mandel accessions.

There is also another variety included in this cluster (C-5), Lillhärjåbygget from Härjedalen, Sweden, where it has been cultivated by the same family at least since the 1850s. It has white flowers and white skin and a tuber shape like Mandel. Unfortunately, we have no knowledge about the morphology of the three Mandel variants I-III in the cluster and can therefore not tell which morphological group described earlier they belong to. We can, however, determine that according to the results from our study, they are all genetically similar to Lillhärjåbygget. This accession was initially classified as a landrace or an improved variety, but our results suggest that it is a landrace.

In the third cluster of this type, C-6, we find Ringerikspotet. The origin of this local variety is unknown, but it has been cultivated for many years in Ringerike in Norway (Veteläinen, 2001). In our study, three different variants kept at NGS, Norway, and one clone with the same name from NordGen, Sweden, were included. The results show that all four clones are genetically identical or very close and if no morphological differences are observed between the three NGS clones it could be considered if all three clones should be kept for future conservation.

### Local material identified as duplicates of improved varieties

Among the 31 clusters of identical or almost identical genotypes (**Table 1**), seven clusters constitute larger groups or pairs of accessions, where one accession is a known improved cultivar, and the others are its duplicates. These clusters are C-1, C-2, C-7, C-10, C-16, C-17, and C-18.

The largest cluster, C-1, contains ten identical or almost identical accessions. This cluster includes the improved variety Arran Victory and its nine duplicates, of which two were thought to be another improved variety, two were considered landraces, and the status of the remaining five was unknown, although their names also suggested that they could be landraces. Arran Victory was bred in 1912 on the Isle of Arran (Scotland) by the breeder David McKelvie and was released on the market in 1918 (Glendinning, 1983). Cluster C-1 is an example of a common pattern observed in the Nordic countries, where a bred variety grown in geographically distant areas receives local names. This applies especially to landraces and older foreign varieties whose difficult and incomprehensible names were replaced by local names, often quite different from the original ones (Hagberth, 1951; Veteläinen, 2001). The available historical sources do not indicate that the cultivation of the Arran Victory variety had a wider range in Nordic countries, unlike the more popular “Arran varieties” such as Arran Banner, Arran Chief, Arran Comrade, or Arran Consul (Øverby, 1929; Hellbo and Esbo, 1942). Nevertheless, Arran Victory is listed among the varieties that were grown both in Norway and Sweden (Potatis i tiden, 2017; Kvann, 2019; NIBIO, 2020a; Fagforum Potet, 2021a). In this cluster, two duplicates of the Arran Victory variety coming from Norway were named Blå Kerrs Pink and Kerrs Pink med blått skall, which could indicate that these two duplicates were thought to be the well-known Kerrs Pink variety where, because of a mutation, the tuber skin changed its color from light red to blue (Kvann, 2019). The Kerrs Pink variety was also analyzed in this study and turned out to be unique and not closely related to these two accessions. Kerrs Pink was the most important table potato variety in Norway for decades. It entered the Norwegian list of varieties in 1953 and is still cultivated (Øverby, 1929; Roer, 1981; Fagforum Potet, 2021b). It is also worth mentioning another accession included in the same cluster called Reyð Epli, coming from the Faroe Islands, which is an autonomous territory of Denmark. Geographically, the Faroe Islands are close to Scotland, where Arran Victory was bred, and it seems likely that trade between these areas also included potatoes. Also, the Finnish accession Karjalan Musta in NordGen’s collection turned out to be identical to Arran Victory. Karjalan Musta from NordGen was also included in an AEGIS project, where duplicates were identified among 379 accessions from eight European collections using 12 SRR markers (Hoekstra and Reid, 2014). In this study, Karjalan Musta showed the same fingerprint as Arran Victory from the British, Irish, and Canadian collections, as well as with other accessions such as Argyll Blue from the United Kingdom, Bleue D’Auvergne from France, Blaue Österreich from Switzerland, Orkney Blue from the United Kingdom, and Skerry Blue from Germany. The morphological characteristics of these duplicates confirm the genotyping results. Therefore, it seems reasonable to conserve only one of these accessions. However, it is worth preserving the names of the duplicates as synonyms of the name Arran Victory and preserving the histories of the individual accessions.

The second-largest cluster, C-2, includes two identical Early Rose accessions, one from NordGen and the other from NGS. The remaining six accessions in this cluster appeared to be duplicates of the Early Rose variety. One of the duplicates was mistakenly taken for another improved variety, and the names of the others suggested that they could be Nordic landraces. Early Rose is an early potato variety bred in 1867 in the USA by the breeder Albert Bresee. This variety gained popularity in the Nordic countries, especially in Denmark and Sweden (Hellbo and Esbo, 1942; Hansen and Nissen, 1945). Early Rose was included on the official list of varieties in Sweden in 1947, and for many years, it was one of the most cultivated early varieties in this country (Osvald, 1965). One of the duplicates in the C-2 cluster was mistakenly given the name of another American variety, namely Early Puritan, also known as Puritan. Early Puritan was the second most popular early potato variety grown in Sweden in the 1950s, together with Early Rose (Osvald, 1940). Hagberth (1951), summarized the results of an inventory of potato varieties grown in Sweden, which tested 349 samples of potatoes from all over the country, submitted under the name Early Rose. Among them, there were only 141 pure samples with the correct variety name. These samples were sometimes mixed with Early Puritan. A large part of the samples was marked with names that were Swedish synonyms of the name Early Rose, and the most popular of them were Rosen, Amerikansk Rosen, Tidig Rosen, Amerikapotatis, Amerikanare and Amerikansk. Veteläinen (2001) also lists 20 different synonyms under which the Early Rose variety was cultivated in Denmark, Finland, Iceland, and Sweden. Many of them refer to the pink color of the tuber or America where the variety comes from. The word “early” in the languages of individual Nordic countries also often appears in these synonyms. It is not surprising therefore that one of the duplicates in the C-2 cluster is called Ameriker. The names of the other duplicates in this cluster, Løgumkloster, and Tranås, are the geographical names of places in Denmark and Sweden from which these accessions originate. Unexpectedly, the Jonsok variety proved to be nearly identical to Early Rose. Jonsok is an early Norwegian variety bred in 1974 from a cross between Saskia and Ulster Prince (Roer, 1981). Consequently, Jonsok was expected to be a unique variety. The name “Jonsok” refers to a Norwegian holiday celebrated on June 24th, commemorating St. John the Baptist and the beginning of summer. Traditionally, the first potatoes of the season are harvested and consumed during Jonsok and similar celebrations in other Nordic countries. It is possible that the Early Rose was mistakenly named Jonsok in this case. If this finding is confirmed, it will be necessary to acquire the correct Jonsok variety for the collection. The Norwegian accession Fljota is named after the old Norwegian word “fljota”, which means “early”. Of the Early Rose variety duplicates, only Løgumkloster has been accepted in the NordGen collection, due to certain morphological differences such as the white skin color, which distinguishes it from the Early Rose accession already present in the collection, as well as due to the interesting history of the accession in Denmark.

The C-10 cluster includes the King Edward VII variety from the United Kingdom and its two Norwegian duplicates, Norska Röda and Raude fra Skjåk, classified as landrace/Improved variety and landrace respectively in the genebanks. The King Edward VII variety, with its unknown parents, was discovered by a gardener from Northumberland. The tubers subsequently reached the hands of breeder John Butler, who introduced the variety to the market in 1902 under the name King Edward VII. Salaman (1926) lists 11 synonyms for this variety. The King Edward VII is still sold in grocery stores across the United Kingdom. Even though King Edward VII was included in the Swedish variety lists in 1947, it remains one of the most popular table potato varieties grown in Sweden (Osvald, 1940; Selga et al., 2022). This variety was also cultivated in Denmark and Norway (Hansen and Nissen, 1945; Roer, 1981). It is significant that King Edward VII does not have as many synonyms as other old foreign varieties grown in the Nordic countries. During an inventory of potato varieties grown in Sweden, among 142 samples marked with the name King Edward VII submitted from across the country, only 2 percent had the incorrect name (Hagberth, 1951). The reason for such a small number of synonyms could be that the name King Edward VII has a familiar sound in the Swedish language, and it is easy to identify by the characteristic appearance and properties of the tubers, such as an oval shape, white skin with shallow red eyes and red areas around the eyes, as well as cream-colored flesh that is mealy when cooked.

In cluster C-16, Granuddspotatis turned out to be a synonym for the old Dutch variety Eigenheimer, bred in 1893. In the Nordic countries, Eigenheimer gained popularity mainly in northern Sweden, in the Norrbotten region. Eigenheimer was included on the Swedish list of varieties in 1947. Granuddspotatis received its name from a place called Granudden, where it was grown, located about 60 kilometers west of the town of Jokkmokk in Swedish Lapland. Other synonyms of the Eigenheimer variety are mentioned in the literature, namely Vaikijaur, Unby, and Guldbolar (Hellbo and Esbo, 1942; Osvald, 1965). The synonym Vaikijaur comes from the town of the same name, also located in the vicinity of Jokkmokk.

The varieties bred by Nordic breeding companies usually have fewer or no synonyms (Veteläinen, 2001). The small number or absence of synonyms in these cases can probably be explained by the fact that the names of varieties bred in the Nordic countries are often rooted in the culture of these countries and are therefore easier to remember, eliminating the need for synonyms. Examples of such varieties are found in clusters C-7, C-17, and C-18. The popular Norwegian variety Beate from 1966 had two synonyms: Kavlås and Olofstorp. The Danish variety Fakse from 2000 had the synonym Per Larsgården. Another Danish variety, Folva, from 1989 had the synonym Ledsjö Gul. One of Folva’s parents is Maris Piper. These accessions are not identical, but the relationship between them is reflected in the dendrogram where both accessions are part of the same cluster (**Figure 2**; **Supplementary Figure S1**).

### Improved varieties conserved under incorrect names

In the case of accessions from the C-23 and C-25 clusters, the results were different from what was expected.

In cluster C-23, two Danish improved varieties, Tylva and Kiva, turned out to be identical. Both varieties were bred by LKF-Vandel, Tylva in 1969 and Kiva in 1970. These two varieties have no common parents. In the dendrogram (**Figure 2**; **Supplementary Figure S1**), they are both grouped together with another Danish variety, Fakse, which has Kiva in its pedigree (van Berloo et al., 2007). Therefore, it can be concluded that both accessions are likely to be Kiva. The accession called Tylva may have been added to the collection under an incorrect name, or there was a mix-up of accessions in the collection. Selga et al. (2022) analyzed Kiva and Tylva from NordGen’s collection using SNP markers. This study also confirmed that both accessions were closely related. Further verification is required, for example, using reference material obtained from a reliable source.

In cluster C-25, two Swedish improved varieties, Maria and Superb, also turned out to be identical. In this case, Superb and Maria group together with the accession HAP 198.4, which is a dihaploid of Maria (**Figure 2**; **Supplementary Figure S1**). Therefore, the correct name is probably Maria. This case should also be verified using reference material.

### Duplicates among blue potatoes

Among the accessions with blue skin and blue-colored flesh, two separate clusters with duplicates, C-8 and C-9, were formed. The C-8 cluster includes the accessions Blå Kartoffel (DDSF), Purple Peruvian, and Vitelotte. Purple Peruvian was added to the NGS collection with the information that the accession comes from Peru, while Vitelotte was added to the LKF-Vandel collection with the information that it is a French landrace. In an AEGIS study where fingerprinting was conducted using microsatellite markers, three Vitelotte accessions from French, German, and Swiss collections were found to be identical to Unbekannte Schwarze from the Czech Republic, Vitelotte Noire from France, Blaue Veltlin from Switzerland, Blaue Peter from the United Kingdom, and Congo from the British and Irish collections (Hoekstra and Reid, 2014). In cluster C-9, the two Congo accessions from NordGen and LKF-Vandel turned out to be identical, as expected. Additionally, the Norwegian accession Svartpotet fra Vegårshei had the same fingerprint. In the aforementioned AEGIS study, Congo from NordGen was found to be identical to accessions such as All Blue, Blaue Schweden, Blue Congo, Blue Salad, McIntosh Black, Russian Blue, Salad Blue, Shetland Beauty, but differed from accessions called Congo from the United Kingdom and Ireland. In Sweden, Congo was also cultivated under the name Svartpotatis, in Denmark under the name Blå kartoffel, in Finland as Läpimusta, in Norway as Sort fra Aasven and Svartpotet, all names including the local name for black or blue. This variety has never had commercial success in the Nordic countries and was grown more as a curiosity (Veteläinen, 2001).

### Landraces cross Nordic borders

Even if not as convenient as seeds, potato tubers have historically been quite easy to bring when moving to other regions. Evidence is also found that old landraces have spread over country borders. When cultivated at a foreign place, it is not inconceivable that by time, the original name changed to a new local one, more or less similar to the original. Duplicate clones with different naming were found in 7 individual clusters in our study (C-4, C-14, C-15, C-19, C-24, C-28, C-29) (**Table 1**) and some of them could be suspected to be examples of this phenomenon. Once again, it is well justified to consider the utility of conserving several duplicate clones in a collection but also to bear in mind potential unique historical narratives connected with the individual clones.

By looking at cluster C-4 it could, not completely unexpected, be confirmed that the accession with the name Rød Kvæfjord (NordGen) is a duplicate variety to Rød Kvæfjording and Gul Kvæfjording, both conserved at NGS. More surprising is, however, the finding that the accession Foula Red (NGS) is a duplicate to these three accessions. Unfortunately, no information about the origin of the clone of Foula red donated to NGS is available. Foula is the name of an island located in the Shetland archipelago of Scotland and according to the European Cultivated Potato Database Foula Red is an advanced cultivar originating in the United Kingdom. However, no release year has been found, and other sources identify Foula Red as a landrace, so this classification is somewhat dubious. If Foula Red is an old cultivar, one explanation could be that it has come to Norway from the United Kingdom and during the years been spread under the wrong name (Rød Kvæfjording). However, as we have identified both Rød and Gul Kvæfjording as well as Rød Kvæfjord with the same genotype in both the Norwegian and the Nordic collection (NordGen) it seems more likely that these clones are the correct ones and that tubers from Rød Kvæfjording on some occasions have been mislabeled as Foula Red. This accession could therefore probably be rejected from the NGS collection.

Another example is the accessions Blå Dalsland and Blå Torpar in cluster C-14. Blå Dalsland is an old landrace which historically has been cultivated in western Sweden as well as in Finland. In Norway a red-skinned variant has been cultivated under the name Nordgårdspotet (Veteläinen, 2001). According to the original documentation at NordGen it was not clear if Blå Torpar was a landrace or an improved variety (**Supplementary Table S1**). It came to NordGen from Gothenburg Botanical Garden which in turn received it from the late Hanna Biljer, a devoted Swedish potato collector. According to her, Blå Torpar is a very old Swedish variety with some connection to the outdoor museum in Skara, Sweden. The varieties Blå Dalsland and Blå Torpar are morphologically similar, and the results from our study confirm that they are indeed duplicates suggesting that Blå Torpar is a landrace with the same origin as Blå Dalsland. This led to the decision to reject Blå Torpar from the collection at NordGen.

Svart Valdres is said to be an established Norwegian synonym name to the variety Bláar Islenskar. The origin of the variety is unknown, but it is known that Bláar Islenskar was among at least three blue skinned varieties that were in cultivation in Iceland in the beginning of the 20^th^ century (Veteläinen, 2001). Svart Valdres is described in a Norwegian publication around the same time (Øverby, 1929). Our study confirms that Svart Valdres from NGS is genetically very close to Bláar Islenskar from NordGen (C-15, **Table 1**). Having these two accessions with different historical names conserved in two different national collections is justified even if they are duplicates as they are connected with their own unique and well documented narratives.

The variety Raudar Islenskar, with the established synonym name Gammal Svensk Röd, is an old local landrace with a possible origin as long ago as 1720 when Jonas Alströmer imported potatoes to Sweden from the Netherlands and France. It can possibly also be one of the first varieties in Iceland 40 years later and has been cultivated in Finland and Norway as well (Veteläinen, 2001). Små Röda is a landrace or improved variety included in the collection at NordGen. According to information from the provider of the accession, it has been cultivated in the Swedish province Småland by his grandfather born 1864. It is also said to have been grown for generations at another farm in the province to which it originally came from England, maybe as the first foreign potato variety entering Sweden. The partly resembling stories of these two accessions together with the results from our study suggests that Små Röda is a duplicate to Raudar Islenskar (C-28). In addition, morphology also partly supports this conclusion and Små Röda has now been rejected from the collection at NordGen.

Cluster C-29 shows that the landrace Tysk Blå and the landrace or improved variety Roslanglänna are genetically identical or very close. Tysk Blå has a probable origin in Germany and has been grown in Sweden, Finland, and Norway, but unfortunately there are no information about donor or origin available for Roslanglänna.

Also, the two clones Gamle Raude fra Aurland and Gammelraude (C-19) as well as Lange’s potet and Tidlig Blå fra Halden (C-24) proved to be duplicates. All four varieties are conserved at NGS.

### Breeding lines identical to Himalaya

One of the clusters, C-21, tells an interesting story. The accession named Himalaya conserved at NordGen proved to be a duplicate of the breeding line I-1039 from the LKF collection. This breeding line was included in a breeding program for late blight resistance at the International Potato Center, CIP, Peru, and came to India as a part of CIP’s work there. I-1039 is cultivated under the names Khumal red 2 and Khumal Rato 2 and was for example found to be the main improved potato variety cultivated in the Bara district in Nepal in 2012 (Kafle and Shah, 2012). I-1039 was developed from Scottish late blight resistance progenitor clones and an unrevealed progenitor, M136–6. This material has two *Solanum* species, *S. phureja* and *S. edinense* included in its pedigree (van Eck et al., 2017). It is also confirmed that it includes the *Ry_sto_* gene from *S. stoloniferum* which makes it highly resistant to Potato Virus Y (PVY) (Herrera et al., 2018). It is possible that an additional species may be included in the genetic heritage of this breeding line because M136–6, the undisclosed ancestor of I-1039, was one of a number of clones with late blight resistance derived from *S. demissum* obtained from Mexico through J.S. Niederhauser of the Rockefeller Foundation in the 1950s (Toxopeus, 1964; Wastie, 1991). I-1039 has resulted in two Danish cultivars, Tivoli, and Liva as well as two other cultivars released in Ecuador (Fripapa 99) and Rwanda (Gikungu) respectively (van Eck et al., 2017).

The origin of Himalaya is somewhat unclear, but the story says that it came to Sweden and Gothenburg Botanical Garden in the beginning of the 1980s with a person that had been traveling in the Himalayas and brought some tubers back home from a potato he found there. The variety did not attract much attention in the botanical garden and the cultivation ceased. However, one of the gardeners brought some tubers to his home garden and continued to grow them there as it proved to be very resistant to potato late blight. In 2007 he gave some tubers back to the botanical garden from which NordGen later received the accession. No morphological comparisons have been performed, but the result from our genetic study indicates that the Indian breeding line conserved at LKF is genetically identical or very close to the accession Himalaya at NordGen. If, and how, the traveler visiting the Himalayas happened to collect tubers from the breeding line I-1039/improved variety Khumal red 2/Khumal Rato 2 we can only speculate about.

The story doesn´t end here. In the same botanical garden, a small seedling was found growing near the Himalaya clone as well as an accession named Kalmar Röd (not included in this study). Both accessions are known for their high resistance to potato late blight. The seedling was rescued by one of the gardeners who took it home and continued to cultivate it for a couple of years under the name Rally. It was shown to possess the same late blight resistance as its suspected mother, Himalaya or Kalmar Röd, and later came back to the botanical garden and was also donated to NordGen. The result from our study confirms that Rally is genetically close to Himalaya even if not as close as I-1039 and Himalaya are to each other. This supports the theory that Rally is a seedling and not a duplicate to Himalaya. However, it cannot be ruled out that the mother could instead be Kalmar Röd which is morphologically close to both Rally and Himalaya. Today both Rally and Himalaya are conserved at NordGen.

## Conclusions

This study used microsatellite markers to identify a large number of unique accessions as well as many duplicates within Nordic potato collections. Accession types, origins, and variety histories were verified, new relationships uncovered and mislabeled accessions were discovered. While the vast majority of studied accessions were unique, a significant number of duplicates were also identified. The largest group was composed of duplicates of old foreign varieties that have received local names and were later conserved under these names. In addition, duplicates of landraces were found in different Nordic countries, suggesting exchange and cultivation of these under local names across the Nordic region. Several identified duplicates likely resulted from human error during collection, handling in genebanks or before they were collected. Accessions with identical or very similar names originating from different collections were, with one exception, found to be duplicates. These findings offer substantial benefits, including reduced collection maintenance costs due to removed duplicates, improved documentation, and facilitated use of the material for plant breeding, research, and education. Furthermore, this study will be valuable for comparing with and corroborating the results from emerging genotyping methods. By providing a well-characterized set of potato accessions with known genetic diversity and clear origin information, the Nordic collections serve as a central reference point for Nordic potato diversity.

## Data Availability

The original contributions presented in the study are publicly available. This data can be found at https://doi.org/10.6084/m9.figshare.26325037.v1.

## References

[B1] Alströmer J. (1777). Potaters plantering, Grundad på Rön och Försök. Kongl. vetenskaps academiens handlingar 38, 246–265.

[B2] Ames M. Spooner D. M. (2008). DNA from herbarium specimens settles a controversy about origins of the European potato. Am. J. Bot. 95, 252–257. doi: 10.3732/ajb.95.2.252 21632349

[B3] Bjor T. (2001). “The history of the potato in Norway,” in Potatis i Norden. En beskrivning av gamla potatissorter bevarade hos Nordiska Genbanken, vol. 55 . Ed. Veteläinen M. (CAL-förlaget AB, Varberg, Sweden).

[B4] Bradshaw J. E. Hackett C. A. Lowe R. McLean K. Stewart H. E. Tierney I.. (2006). Detection of a quantitative trait locus for both foliage and tuber resistance to late blight Phytophthora infestans (Mont.) de Bary on chromosome 4 of a dihaploid potato clone (Solanum tuberosum subsp. tuberosum). Theor. Appl. Genet. 113, 943–951. doi: 10.1007/s00122-006-0353-8 16845519

[B5] Doyle J. J. Doyle J. L. (1987). A rapid DNA isolation procedure for small quantities of fresh leaf tissue. Phytochem. Bull. 19, 11–15.

[B6] Duan Y. Liu J. Xu J. Bian C. Duan S. Pang W.. (2019). DNA fingerprinting and genetic diversity analysis with simple sequence repeat markers of 217 potato cultivars (*Solanum tuberosum* L.) in China. Am. J. Potato Res. 96, 21–32. doi: 10.1007/s12230-018-9685-6

[B7] Erjefält L. (2001). “The history of the potato in Sweden,” in Potatis i Norden. En beskrivning av gamla potatissorter bevarade hos Nordiska Genbanken. Ed. Veteläinen M. (CAL-förlaget AB, Varberg, Sweden), 65–66.

[B8] Fagforum Potet (2021a). Arran victory. Available online at: https://potet.no/produkter/arran-victory (Accessed March 03, 2024).

[B9] Fagforum Potet (2021b). Kerrs Pink. Available online at: https://potet.no/produkter/kerrs-pink (Accessed March 03, 2024).

[B10] Feingold S. Lloyd J. Norero N. Bonierbale M. Lorenzen J. (2005). Mapping and characterization of new EST-derived microsatellites for potato (Solanum tuberosum L.). Theor. Appl. Genet. 111, 456–466. doi: 10.1007/s00122-005-2028-2 15942755

[B11] Ghislain M. Núñez J. del Rosario Herrera M. Pignataro J. Guzman F. Bonierbale M.. (2009). Robust and highly informative microsatellite-based genetic identity kit for potato. Mol. Breed. 23, 377–388. doi: 10.1007/s11032-008-9240-0

[B12] Ghislain M. Spooner D. M. Rodríguez F. Villamón F. Núñez J. Vásquez C.. (2004). Selection of highly informative and user-friendly microsatellites (SSRs) for genotyping of cultivated potato. Theor. Appl. Genet. 108, 881–890. doi: 10.1007/s00122-003-1494-7 14647900

[B13] Glendinning D. R. (1983). Potato introductions and breeding up to the early 20th century. New Phytol. 94, 479–505. doi: 10.1111/j.1469-8137.1983.tb03460.x

[B14] Hagberth N. O. (1951). Potatissorterna i sverige. Potato varieties in Sweden. R. Agr. Col. Sweden Inst. Plant Husb. Publ. 5, 194.

[B15] Hagenblad J. Aloisi K. Marum P. Öhlund L. Solberg S. Ø. Asdal Å.. (2023). Limited genetic changes observed during in *situ* and *ex situ* conservation in Nordic populations of red clover (*Trifolium pratense*). Front. Plant Sci. Sec. Plant Breed. 14. doi: 10.3389/fpls.2023.1233838 PMC10445542 37621888

[B16] Hansen H. R. Nissen M. (1945). Beskrivelse af kartoffelsorter, dyrket i Danmark. Tidsskrift Planteavl 49, 559–663.

[B17] Hawkes J. G. (1990). The Potato: Evolution, Biodiversity and Genetic Resources (London: Belhaven Press).

[B18] Hawkes J. G. Francisco-Ortega J. (1992). The potato in Spain during the late 16^th^ century. Econ. Bot. 46, 86–97. doi: 10.1007/BF02985257

[B19] Hawkes J. G. Francisco-Ortega J. (1993). The early history of the potato in Europe. Euphytica 70, 1–7. doi: 10.1007/BF00029633

[B20] Hellbo E. Esbo H. (1942). Våra potatissorter. Systematisk Behandling (Stockholm: Lantbruksförbundets tidskriftsaktiebolag).

[B21] Herrera M. D. R. Vidalon L. J. Montenegro J. D. Riccio C. Guzman F. Bartolini I.. (2018). Molecular and genetic characterization of the Ry_adg_ locus on chromosome XI from Andigena potatoes conferring extreme resistance to potato virus Y. Theor. Appl. Genet. 131, 1925–1938. doi: 10.1007/s00122-018-3123-5 29855674 PMC6096621

[B22] Hoekstra R. Reid A. (2014). Final report of the AEGIS project: identification of old potato clones having unreliable variety names by means of fingerprinting using microsatellite (SSR) markers to assist in setting up the AEGIS collection for potato cultivars. Available online at: https://www.ecpgr.cgiar.org/fileadmin/templates/ecpgr.org/upload/PROJECT_REPORTS/Potato_project_AEGIS_Grant_Final_report.pdf (Accessed March 03, 2024).

[B23] Kafle B. Shah P. (2012). Adoption of improved potato varieties in Nepal: A case of Bara district. J. Agric. Sci. – Sri Lanka 7, 14–22. doi: 10.4038/jas.v7i1.4063

[B24] Kawchuk L. M. Lynch D. R. Thomas J. Penner B. Sillito D. Kulcsar F. (1996). Characterization of Solanum tuberosum simple sequence repeats and application to potato cultivar identification. Am. Potato J. 73, 325–335. doi: 10.1007/BF02849164

[B25] Kishine M. Tsutsumi K. Kitta K. (2017). A set of tetra-nucleotide core motif SSR markers for efficient identification of potato (*Solanum tuberosum*) cultivars. Breed Sci. 67, 544–547. doi: 10.1270/jsbbs.17066 29398950 PMC5790051

[B26] Kørup Sørensen K. Kirk H. G. Emmersen J. (2007). “Microsatellites used for estimation of relationship between potato breeding clones,” in Plant Biotech Denmark. Annual meeting 2007: Faculty of Life Sciences, University of Copenhagen, (Copenhagen: PBD Plant Biotech Denmark).

[B27] Kvann (2019). Historiske potetsorter dyrket i Norge. Available online at: https://kvann.no/wp-content/uploads/2019/11/20190316_Sortsbeskrivelse_poteter_utsendelse_vaaren2019.pdf (Accessed March 03, 2024).

[B28] Kyrre H. (1938). Kartoffelens krønike: en kulturhistorisk studie (København: Gad).

[B29] McNeill W. H. (1999). How the potato changed the world’s history. Soc Res. 66, 67–83. 22416329

[B30] Milbourne D. Meyer R. Collins A. Ramsay A. Gebhardt C. Waugh R. (1998). Isolation, characterization and mapping of simple sequence repeat loci in potato. Mol. Gen. Genet. 259, 233–245. doi: 10.1007/s004380050809 9749666

[B31] Muhinyuza J. B. Shimelis H. Melis R. Sibiya J. Gahakwa D. Nzaramba M. (2015). Assessment of genetic relationship among promising potato genotypes in Rwanda using SSR markers. Aust. J. Crop Sci. 9, 696–700.

[B32] NIBIO (2020a). Potet. Arran victory. Available online at: https://www.nibio.no/tema/mat/plantegenetiske-ressurser/nytteplanter-i-norge/jordbruksplanter/potet/arran-victory (Accessed March 03, 2024).

[B33] NIBIO (2020b). Potet. Mandelpotet. Available online at: https://nibio.no/tema/mat/plantegenetiske-ressurser/nytteplanter-i-norge/jordbruksplanter/potet/mandelpotet (Accessed March 03, 2024).

[B34] Osvald H. (1940). Potatis (Stockholm: Kooperativa Förbundets bokförlag).

[B35] Osvald H. (1965). Potatisen: odlingshistoria och användning (Uppsala: Almqvist och Wiksells boktryckeri aktiebolag).

[B36] Øverby G. (1929). Morfologiske sortskarakterer hos potet. Bidrag til beskrivelse av 98 potetsorter Vol. 9 (Ås: Meldinger fra Norges Landbrukshøiskole), 429–527.

[B37] Potatis i tiden (2017). Sorter. Available online at: https://potatisitiden.se/sorter/ (Accessed March 03, 2024).

[B38] Provan J. Powell W. Waugh R. (1996). Microsatellite analysis of relationships within cultivated potato (Solanum tuberosum). Theor. Appl. Genet. 92, 1078–1084. doi: 10.1007/BF00224052 24166639

[B39] Reid A. Kerr E. M. (2007). A rapid simple sequence repeat (SSR)-based identification method for potato cultivars. Plant Genet. Resour. 5, 7–13. doi: 10.1017/S1479262107192133

[B40] Ríos D. Ghislain M. Rodríguez F. Spooner D. M. (2007). What is the origin of the European potato? Evidence from Canary Island landraces. Crop Sci. 47, 1270–1280. doi: 10.2135/CROPSCI2006.05.0336

[B41] Roer L. (1981). Beskrivelse av potetsorter (Ås: Statens planteavlsråd).

[B42] Roer L. (1992). Nordisk genbank. Arbeidet med sikring av verdifullt potetmateriale. Fagnytt hagebruk 12 (Ås: Statens fagtjenste for landbruket).

[B43] Salaman R. N. (1926). Potato varieties (Cambridge: Cambridge University Press).

[B44] Selga C. Chrominski P. Carlson-Nilsson U. Andersson M. Chawade A. Ortiz R. (2022). Diversity and population structure of Nordic potato cultivars and breeding clones. BMC Plant Biol. 22, 350. doi: 10.1186/s12870-022-03726-2 35850617 PMC9290215

[B45] Spanoghe M. Marique T. Nirsha A. Esnault F. Lanterbecq D. (2022). Genetic diversity trends in the cultivated potato: A spatiotemporal overview. Biology 11, 604. doi: 10.3390/biology11040604 35453803 PMC9026384

[B46] Spooner D. M. Núñez J. Rodriguez F. Naik P. S. Ghislain M. (2005). Nuclear and chloroplast DNA reassessment of the origin of Indian potato varieties and its implications for the origin of the early European potato. Theor. Appl. Genet. 110, 1020–1026. doi: 10.1007/s00122-004-1917-0 15754208

[B47] Swederus M. B. (1877). Botaniska trädgården i Upsala 1655–1807: ett bidrag till den svenska naturforskningens historia (Falun: Falu Boktryckeri-Aktiebolag).

[B48] Szajko K. Chrzanowska M. Witek K. Strzelczyk-Żyta D. Zagórska H. Gebhardt C.. (2008). The novel gene *Ny-1* on potato chromosome IX confers hypersensitive resistance to *Potato virus Y* and is an alternative to *Ry* genes in potato breeding for PVY resistance. Theor. Appl. Genet. 116, 297–303. doi: 10.1007/s00122-007-0667-1 17985110 PMC2755788

[B49] Tamura K. Stecher G. Kumar S. (2021). MEGA11: molecular evolutionary genetics analysis version 11. Mol. Biol. Evol. 38, 3022–3027. doi: 10.1093/molbev/msab120 33892491 PMC8233496

[B50] Tamura K. Stecher G. Peterson D. Filipski A. Kumar S. (2013). MEGA6: molecular evolutionary genetics analysis version 6.0. Mol. Biol. Evol. 30, 2725–2729. doi: 10.1093/molbev/mst197 24132122 PMC3840312

[B51] Tolstrup K. (2001). “The potato in Denmark,” in Potatis i Norden. En beskrivning av gamla Potatissorter Bevarade hos Nordiska Genbanken. Ed. Veteläinen M. (CAL-förlaget AB, Varberg, Sweden), 32–35.

[B52] Toxopeus H. J. (1964). Treasure-digging for blight resistance in potatoes. Euphytica 13, 206–222. doi: 10.1007/BF00023099

[B53] Umaerus M. (1989). “True potato seed,” in Reprinted in 1989 from: EAPR Proceedings, 10th Triennial Conference of the European Association for Potato Research, Aalborg, Denmark, July 26-31, 1987 (Lima: International Potato Center).

[B54] van Berloo R. Hutten R. C. B. van Eck H. J. Visser R. G. F. (2007). An online potato pedigree database resource. Potato Res. 50, 45–57. doi: 10.1007/s11540-007-9028-3

[B55] van Eck H. J. Vos P. G. Valkonen J. P. T. Uitdewilligen J. G. M. L. Lensing H. de Vetten N.. (2017). Graphical genotyping as a method to map *Ny_(o,n) sto_ * and *Gpa5* using a reference panel of tetraploid potato cultivars. Theor. Appl. Genet. 130, 515–528. doi: 10.1007/s00122-016-2831-y 27872942 PMC5315735

[B56] Varis E. (2001). “The potato in Finland from the past to the present,” in Potatis i Norden. En beskrivning av gamla potatissorter bevarade hos Nordiska Genbanken. Ed. Veteläinen M. (CAL-förlaget AB, Varberg, Sweden), 44–45.

[B57] Veteläinen M. (Ed.) (2001). Potatis i Norden. En beskrivning av gamla potatissorter bevarade hos Nordiska Genbanken (Varberg: CAL-förlaget AB).

[B58] Veteläinen M. Gammelgård E. Valkonen J. P. T. (2005). Diversity of Nordic landrace potatoes (*Solanum tuberosum* L.) revealed by AFLPs and morphological characters. Genet. Resour. Crop Evol. 52, 999–1010. doi: 10.1007/s10722-003-6129-y

[B59] Wastie R. L. (1991). “Breeding for resistance,” in *Phytophthora infestans*, the cause of Late Blight of Potato. Advances in Plant Pathology, vol. 7 . Eds. Ingram D. S. Wiliams D. S. (Academic Press Ltd., London), 193–224.

[B60] Yndgaard F. Solberg S. Ø. (2019). 40 years of Nordic Collaboration in Plant Genetic Resources (Alnarp: Nordic Genetic Resources Center).

