# Supplementary material for: Genetic markers identify duplicates in Nordic potato collections
Source: Front Plant Sci. 2024 Aug 26;15:1405314. doi: 10.3389/fpls.2024.1405314 (PMC11381411; doi:10.3389/fpls.2024.1405314)
Supplement: Supplementary file 1 [file DataSheet1.pdf]

Supplementary tables

Table S1. The 198 potato accessions included in this study. Information on accession name as given in the collection where the accession is conserved, the collection (LKF = Danish Potato Breeding Foundation in Vandel; NGB = Nordic Genetic Resource Centre (NordGen); NGS = Norwegian Genetic Resource Centre), accession type, pedigree if available, origin country, release year for varieties and accession number for the accessions from NordGen and NGS.

Accession name	Collection	Accession type (as of 2013)	Pedigree	Origin country	Release year	Accession number
**92-BSI-702**	LKF	Breeding line	Alex x Sava	Denmark		
**HAP 198.4**	LKF	Breeding line	not available	Denmark		
**I-1039**	LKF	Breeding line	not available	India		
**Lü 56.220/94**	LKF	Breeding line	Li 1005/47 (=Sabina x Ackersegen) x Lü 55.428/3 N	Denmark		
**Alex**	LKF	Improved variety	C1 22 x SVP VTN 62 33 3	Denmark	1997	
**Appell**	LKF	Improved variety	Escort x Annika	Sweden	1997	
**Bintje**	LKF	Improved variety	Munstersen x Fransen	Netherlands	1910	
**Bruse**	LKF	Improved variety	Lady Rosetta x Hedemarkspotet	Norway	2001	
**Danva**	LKF	Improved variety	Amia (Amigo) x *S. vernei* hybrid	Denmark	1981	
**Desiree**	LKF	Improved variety	Urgenta x Depesche	Netherlands	1962	
**Fakse**	LKF	Improved variety	Lutetia x Asva	Denmark	2000	
**Folva**	LKF	Improved variety	Miranda x Maris Piper	Denmark	1989	
**Juli**	LKF	Improved variety	Josef Rigault x Pflückmaus	Germany	1891	
**Kaptah**	LKF	Improved variety	Robusta x Flava	Denmark	1962	
**King Edward VII**	LKF	Improved variety	Magnum Bonum x Beauty of Hebron	UK	1902	
**Kiva**	LKF	Improved variety	Lü 56.220/94 x Dr McIntosh	Denmark	1970	
**Magnum Bonum**	LKF	Improved variety	Early Rose x Paterson's Victoria	UK	1876	
**Maria**	LKF	Improved variety	Falke x Cayuga	Sweden	1972	

**Maris Piper**	LKF	Improved variety	(*S. andigena* x Ulster Knight) x (Arran Cairn x Herald)	Ireland	1963	
**Minea**	LKF	Improved variety	Arran Pilot x Menominee	Denmark	1962	
**Octavia**	LKF	Improved variety	Lü 56.220/94 x Asparges (Ratte)	Denmark	1970	
**Oleva**	LKF	Improved variety	N70-AEJ-6= (Minsand x *S. vernei* hybrid) x Posmo	Denmark	1990	
**Posmo**	LKF	Improved variety	Erdkraft x Gineke	Denmark	1978	
**Reichskanzler**	LKF	Improved variety	Seed x Daber	Germany	1886	
**Robijn**	LKF	Improved variety	Rode Star x Preferent	Netherlands	1926	
**Rosva**	LKF	Improved variety	Bintje x Dr McIntosh	Denmark	1972	
**Russet Burbank LKF**	LKF	Improved variety	Mutant of Burbank	USA	1908	
**Semlo LKF**	LKF	Improved variety	Bintje x Gabriela	Denmark	1978	
**Sharpes Express (Sydens Dronning)**	LKF	Improved variety	not available	UK	1900	
**Tertus**	LKF	Improved variety	Lü 56.220/94 x Asparges	Denmark	1975	
**Up to date**	LKF	Improved variety	Paterson's Victoria x Blue Don	UK	1894	
**White Lady**	LKF	Improved variety	KE 40 x 71 17 6	Hungary	1994	
**Æggeblomme LKF**	LKF	Landrace		Denmark		
**Asparges LKF**	LKF	Landrace		Denmark		
**Blå Kartoffel (DDSF)**	LKF	Landrace		Denmark		
**Brønderslev kartoffel**	LKF	Landrace		Denmark		
**Congo LKF**	LKF	Landrace		Sweden		
**Gullauge LKF**	LKF	Landrace		Norway		
**Kefermarkter Zuchtstamm**	LKF	Landrace		Austria		
**Violettfleischige**	LKF	Landrace		Germany		
**Vitelotte**	LKF	Landrace		France		
**Webb's Tidlig**	LKF	Landrace		Denmark		
**Weinberger Blaue**	LKF	Landrace		Austria		
**N 73-22-2**	NGB	Breeding line	Pimpernel x Amva	Norway		NGB 3467
**N-73-20-11**	NGB	Breeding line	Troll x Amva	Norway		NGB 3227

**P 117**	NGB	Breeding line	Frühperle x Gabriela/Mexico/	Sweden		NGB 3252
**P 141**	NGB	Breeding line	Anita/Mexico/ x Hansa	Sweden		NGB 3254
**P 325**	NGB	Breeding line	Elsa x EYK 5 DT3/Reddick/	Sweden		NGB 3251
**P 40**	NGB	Breeding line	Parnassia x (EYK 169 DT3/Reddick/ x Elsa)	Sweden		NGB 3253
**Y-67-20-40**	NGB	Breeding line	(Grazilia x nummersort) x (Falke x Aquila-1)	Norway		NGB 3226
**Annika**	NGB	Improved variety	Carolina x Sv 68113	Sweden	1988	NGB 21725
**Åspotet_NGB**	NGB	Improved variety	Hindenburg x Centifolia	Norway	1932	NGB 3225
**Birgitta**	NGB	Improved variety	Magnum Bonum x Badera	Sweden	1924	NGB 3160
**Columbo**	NGB	Improved variety	Sv 76127 x Matilda	Sweden	1996	NGB 21727
**Early Rose NGB**	NGB	Improved variety	Seedling of Garnet Chili	USA	1867	NGB 3276
**Elin**	NGB	Improved variety	MPI 54 4129 288 x CPC 2070 59	Sweden	1984	NGB 3390
**Elsa**	NGB	Improved variety	Alpha x Sv 19149	Sweden	1948	NGB 3192
**Eva**	NGB	Improved variety	King George V x Mittelfrühe	Sweden	1950	NGB 3125
**Hankkijan Tuomas**	NGB	Improved variety	Amyla x Horsa	Finland	1975	NGB 3257
**Hårek NGB**	NGB	Improved variety	Pimpernel x Beate	Norge	1985	NGB 3468
**Jaakko**	NGB	Improved variety	Eigenheimer x Goldwährung	Finland	1951	NGB 3396
**Jøssing NGB**	NGB	Improved variety	Louis Botha x Jubel	Norway	1945	NGB 3395
**Karna**	NGB	Improved variety	Unica x Athene	Sweden	1945	NGB 3246
**Koto**	NGB	Improved variety	Eigenheimer x Kalev	Finland	1961	NGB 3255
**Marius NGB**	NGB	Improved variety	Goodrich x Reichskanzler	Poland	1893	NGB 3100
**Pito**	NGB	Improved variety	Golden Wonder x Ella	Finland	1964	NGB 3493
**Prestkvern NGB**	NGB	Improved variety	Kerrs Pink x Jubel	Norway	1938	NGB 3394
**Semlo NGB**	NGB	Improved variety	Bintje x Gabriela	Denmark	1978	NGB 3200
**Silla**	NGB	Improved variety	Desiree x (Lü 56.220/94 x Dr McIntosh)	Sweden	1983	NGB 3392
**Stina**	NGB	Improved variety	Amaryl x 5110 (=Parnassia x D 394)	Sweden	1976	NGB 3228
**Superb**	NGB	Improved variety	Solanda x Matilda	Sweden	2003	NGB 23555
**Tammiston Aikainen**	NGB	Improved variety	Up to date x Prof. Edler	Sweden	1930	NGB 3223
**Tylva**	NGB	Improved variety	Dianella x Börger 879/49	Denmark	1969	NGB 3492
**Valtti**	NGB	Improved variety	Frühnudel (=Nuutti) x Elsa	Finland	1965	NGB 3070

**Vestar**	NGB	Improved variety	Pimpernel x (Doon Star x Åspotet-1006)	Norway	1972	NGB 3224
**Veto**	NGB	Improved variety	Aquila x Rosafolia	Finland	1968	NGB 3256
**Æggeblomme NGB**	NGB	Landrace		Denmark		NGB 3054
**Backpotatis**	NGB	Landrace		Sweden		NGB 3414
**Björna**	NGB	Landrace		Sweden		NGB 3207
**Blå Dalsland**	NGB	Landrace		Sweden		NGB 3035
**Blå Mandel**	NGB	Landrace		Sweden		NGB 3230
**Blaar Islenskar**	NGB	Landrace		Iceland		NGB 3304
**Congo (SWE)**	NGB	Landrace		Sweden		NGB 3312
**Fjellfinn**	NGB	Landrace		Norway		NGB 3212
**Gular Islenskar**	NGB	Landrace		Iceland		NGB 3147
**Jämtlands Vit**	NGB	Landrace		Sweden		NGB 3176
**Karjalan Musta**	NGB	Landrace		Finland		NGB 3375
**Köttpotatis**	NGB	Landrace		Sweden		NGB 3300
**Lang Svenske**	NGB	Landrace		Norway		NGB 3168
**Lange Røde**	NGB	Landrace		Denmark		NGB 3048
**Leksands Vit**	NGB	Landrace		Sweden		NGB 3013
**Lemin Punanen**	NGB	Landrace		Finland		NGB 3267
**Rättviks Röd**	NGB	Landrace		Sweden		NGB 3019
**Raudar Islenskar (Gammal Svensk Röd)**	NGB	Landrace		Iceland		NGB 3149
**Ringerikspotet NGB**	NGB	Landrace		Norway		NGB 3494
**Rød Kvæfjord**	NGB	Landrace		Norway		NGB 3170
**Röda Krokar**	NGB	Landrace		Sweden		NGB 3193
**Rödbrokig Svensk**	NGB	Landrace		Sweden		NGB 3062
**Sort IV fra Aukrust**	NGB	Landrace		Norge		NGB 3210
**Sparrispotatis**	NGB	Landrace		Sweden		NGB 3007
**Tärendö Karl Krekula**	NGB	Landrace		Sweden		NGB 3502
**Tysk Blå**	NGB	Landrace		Sweden		NGB 3155

**Ameriker**	NGB	Landrace/Improved variety		Sweden		NGB 23567
**Bjärsgård**	NGB	Landrace/Improved variety		Sweden		NGB 23561
**Blå Märta**	NGB	Landrace/Improved variety		Sweden		NGB 20400
**Blå Torpar**	NGB	Landrace/Improved variety		Sweden		NGB 23556
**Bredegård**	NGB	Landrace/Improved variety		Sweden		NGB 23564
**Granuddspotatis**	NGB	Landrace/Improved variety		Sweden		NGB 23640
**Gul Potatis**	NGB	Landrace/Improved variety		Sweden		NGB 20399
**Gullan Lerdala**	NGB	Landrace/Improved variety		Sweden		NGB 23549
**Gusten Älgarås**	NGB	Landrace/Improved variety		Sweden		NGB 23559
**Hannika**	NGB	Landrace/Improved variety		Sweden		NGB 23562
**Himalaya**	NGB	Landrace/Improved variety		Nepal		NGB 23571
**Kavlås**	NGB	Landrace/Improved variety		Sweden		NGB 23560
**Ledsjö Gul**	NGB	Landrace/Improved variety		Sweden		NGB 23552
**Lillhärjåbygget**	NGB	Landrace/Improved variety		Sweden		NGB 20395
**Løgumkloster**	NGB	Landrace/Improved variety		Denmark		NGB 20401
**Luröpotatis**	NGB	Landrace/Improved variety		Sweden		NGB 24297
**Mätsälä**	NGB	Landrace/Improved variety		Finland		NGB 24298
**Mozart från Siene**	NGB	Landrace/Improved variety		Sweden		NGB 23550
**Närke Vit**	NGB	Landrace/Improved variety		Sweden		NGB 23554
**Norska Röda**	NGB	Landrace/Improved variety		Sweden		NGB 23566
**Olofstorp**	NGB	Landrace/Improved variety		Sweden		NGB 23551
**Pålle Kättilstorp**	NGB	Landrace/Improved variety		Sweden		NGB 23557
**Per Larsgården**	NGB	Landrace/Improved variety		Sweden		NGB 23558
**Purpur**	NGB	Landrace/Improved variety		Sweden		NGB 20398
**Rally**	NGB	Landrace/Improved variety		Sweden		NGB 24281
**Reyð Epli**	NGB	Landrace/Improved variety		Faroe Islands		NGB 20404
**Röd Mandel**	NGB	Landrace/Improved variety		Sweden		NGB 24296
**Roslanglänna**	NGB	Landrace/Improved variety		Sweden		NGB 20402
**Små Röda**	NGB	Landrace/Improved variety		Sweden		NGB 21097

**Tranås**	NGB	Landrace/Improved variety		Sweden		NGB 24775
**Vallgren Falbygden**	NGB	Landrace/Improved variety		Sweden		NGB 23563
**Abundance/Ebbedens**	NGS	Improved variety	Magnum Bonum x Fox's seedling	UK	1886	506
**Aksel**	NGS	Improved variety	Snøgg x N73-20-11	Norway	2000	235
**Arran Victory**	NGS	Improved variety	Seedling of Sutton's Abundance	UK	1918	217
**Åspotet NGS**	NGS	Improved variety	Hindenburg x Centifolia	Norway	1932	127
**Beate**	NGS	Improved variety	Carnea x (Doon Star x Åspotet 737)	Norway	1966	236
**Brage**	NGS	Improved variety	Ottar x Pentland Javelin	Norway	1988	237
**British Queen**	NGS	Improved variety	Paterson's Victoria x Old Blue Don	UK	1894	388
**Early Puritan**	NGS	Improved variety	Seedling of Beauty of Hebron	USA	1888	123
**Early Rose NGS**	NGS	Improved variety	Seedling of Garnet Chili	USA	1867	264
**Eigenheimer**	NGS	Improved variety	Blauwe Reuzen x Fransen	Netherlands	1895	213
**Garnet Chili**	NGS	Improved variety	Open pollinated seedling of Rough Purple Chili	USA	1857	160
**Grom**	NGS	Improved variety	Early Puritan x Rutt	Norway	1999	240
**Hårek NGS**	NGS	Improved variety	Pimpernel x Beate	Norway	1985	29
**Iverpotet/Smaragd**	NGS	Improved variety	Bella x Rheinhort	Germany	1968	508
**Jonsok**	NGS	Improved variety	Saskia x Ulster Prince	Norway	1974	224
**Jøssing NGS**	NGS	Improved variety	Louis Botha x Jubel	Norway	1945	220
**Kerrs Pink**	NGS	Improved variety	Fortyfold x Smith's Early	UK	1907	244
**Laila**	NGS	Improved variety	Pimpernel x (Doon Star x Åspotet-737)	Norway	1969	246
**Marius II**	NGS	Improved variety	Goodrich x Reichskanzler	Poland	1893	389
**Olsok**	NGS	Improved variety	Kerrs Pink x Venus	Norway	1965	221
**Ottar**	NGS	Improved variety	Dore x Pimpernel	Norway	1974	252
**Peik**	NGS	Improved variety	X-65-18-17 x Saturna	Norway	1984	463
**Pimpernel**	NGS	Improved variety	Populair x [Bravo x Alpha (=Mulder K 101)]	Netherlands	1953	463
**Prestkvern NGS**	NGS	Improved variety	Kerrs Pink x Jubel	Norway	1938	267
**Russet Burbank NGS**	NGS	Improved variety	Mutant of Burbank	USA	1908	36
**Rutt**	NGS	Improved variety	Laila x Alcmaria	Norway	1982	256
**Sharps Express**	NGS	Improved variety	not available	UK	1900	40

**Shetland Black**	NGS	Improved variety	not available	UK	1923	398
**Troll**	NGS	Improved variety	(Dore x Pimpernel) x (Falke x Aquila)	Norway	1981	260
**Asparges NGS**	NGS	Landrace		Norway		270
**Fljota**	NGS	Landrace		Norway		390
**Foula Red**	NGS	Landrace		Norway		392
**Gamle Raude fra Aurland**	NGS	Landrace		Norway		394
**Gjernespotet**	NGS	Landrace		Norway		561
**Gul Kvæfjording**	NGS	Landrace		Norway		316
**Hjelvik**	NGS	Landrace		Norway		397
**Hroar Dege**	NGS	Landrace		Norway		507
**Ingeleivseple**	NGS	Landrace		Norway		395
**Lange's potet**	NGS	Landrace		Norway		399
**Purple Peruvian**	NGS	Landrace		Peru		458
**Raude fra Skjåk**	NGS	Landrace		Norway		393
**Svart Valdres**	NGS	Landrace		Norway		302
**Svart/Blå fra Skjåk**	NGS	Landrace		Norway		391
**Svartpotet fra Vegårshei**	NGS	Landrace		Norway		50
**Sverre**	NGS	Landrace		Norway		510
**Tidlig Blå fra Halden**	NGS	Landrace		Norway		682
**Tromøypotet**	NGS	Landrace		Norway		300
**Blå fra Onsøy**	NGS	Landrace/Improved variety		Norway		396
**Blå Kerrs Pink**	NGS	Landrace/Improved variety		Norway		67
**Buddhisten fra Snåsa**	NGS	Landrace/Improved variety		Chile		509
**Gammelraude**	NGS	Landrace/Improved variety		Norway		505
**Gullauge Gul variant II**	NGS	Landrace/Improved variety		Norway		299
**Gullauge Rød**	NGS	Landrace/Improved variety		Norway		199
**Gullauge Gul variant I**	NGS	Landrace/Improved variety		Norway		241

**Kerrs Pink med blått skall**	NGS	Landrace/Improved variety		Norway		504
**Mandel variant I (klon 1)**	NGS	Landrace/Improved variety		Norway		462
**Mandel variant II (Ekrann)**	NGS	Landrace/Improved variety		Norway		45
**Mandel variant III (klon 6)**	NGS	Landrace/Improved variety		Norway		461
**Ringerikspotet variant I**	NGS	Landrace/Improved variety		Norway		255
**Ringerikspotet variant II**	NGS	Landrace/Improved variety		Norway		265
**Ringerikspotet variant III**	NGS	Landrace/Improved variety		Norway		449
**Rød Kvæfjording**	NGS	Landrace/Improved variety		Norway		317
**Rosenpotet**	NGS	Landrace/Improved variety		Norway		164
**Rosenring**	NGS	Landrace/Improved variety		Norway		294
**Truls**	NGS	Landrace/Improved variety		Norway		503

Table S2. The 62 microsatellite markers (and one SCAR marker) used in the study, with the reference to where they were first published, the repeat number, the range of allele sizes in base pairs, and the number of alleles identified in the investigated dataset.

**Marker**	**Reference**	**Repeat**	**Allele size range (bp)**	**No. alleles**
LeatpacA	Milbourne et al., 1998	(TA)7	225 - 232	4
SC895	Szajko et al., 2008	SCAR marker	na	na
SSR1	Kawchuk et al. 1996	(TCAC)n	201 - 229	12
StI004	Feingold et al., 2005	(AAG)n	70 - 101	10
StI009	Feingold et al., 2005	(AGC)n(AAC)n	261 - 285	9
StI013	Feingold et al., 2005	(ACC)n	96 - 307	9
StI014	Feingold et al., 2005	(TGG)n(AGG)n	113 - 128	7
StI015	Feingold et al., 2005	(AT)n(AG)n(AT)n	180 - 253	19
StI016	Feingold et al., 2005	(CCT)n	286 - 312	10
StI018	Feingold et al., 2005	(ATA)n	161 - 191	6
StI029	Feingold et al., 2005	(CA)imp + (TC)imp	122 - 154	8
StI032	Feingold et al., 2005	(GGA)n	107 - 126	7
StI043	Feingold et al., 2005	(AAC)imp	126 - 141	6
StI047	Feingold et al., 2005	(TAA)n	127 - 145	7
StI051	Feingold et al., 2005	(TA)n	148 - 184	12
StI053	Feingold et al., 2005	(AT)imp	106 - 158	11
StI055	Feingold et al., 2005	(AAG)n	213 - 239	6
StI058	Feingold et al., 2005	(TA)n	70 - 121	13
StI060	Feingold et al., 2005	(ATA)n	154 - 169	4
STM0003	Milbourne et al., 1998	(AC)9(AT)9	107 - 165	11
STM0019	Milbourne et al., 1998	(AT)7(GT)10(AT)4(GT)5(GC)4(GT)4	85 - 229	12
STM0028	Milbourne et al., 1998	(AC)12(AT)5(AG)8	118 - 142	10
STM0030	Milbourne et al., 1998	compound((GT/GC)GT)8	133 - 165	10
STM0031	Milbourne et al., 1998	(AC)5…(AC)3(GCAC).(AC)2(GCAC)2	49 - 199	18
STM0037	Milbourne et al., 1998	(TC)5(AC)6AA..(AC)7(AT)4	65 - 83	8
STM0051	Milbourne et al., 1998	(AC)7…(AC)7(AT)4	105 - 111	4
STM0052	Milbourne et al., 1998	(AC)5…(AC)6.(AC)16	100 - 160	9
STM1016	Milbourne et al., 1998	(TCT)9	238 - 264	9
STM1024	Milbourne et al., 1998	(TTG)6	134 - 154	7
STM1049	Milbourne et al., 1998	(ATA)6	181 - 202	6
STM1051	Milbourne et al., 1998	(TAT)4TTT(TAT)7	182 - 252	12
STM1052	Milbourne et al., 1998	(AT)14GT(AT)4(GT)6	207 - 252	8
STM1058	Milbourne et al., 1998	(ATT)5	103 - 124	6
STM1102	Milbourne et al., 1998	(TA)8	143 - 198	6
STM1104	Milbourne et al., 1998	(TCT)5	167 - 183	8
STM1106	Milbourne et al., 1998	(ATT)13	145 - 191	8
STM2005	Milbourne et al., 1998	(CTGTTG)3	147 - 191	5
STM2022	Milbourne et al., 1998	(CAA)3...(CAA)3	175 - 229	4
STM2028	Milbourne et al., 1998	(TAC)5...(CT)5	242 - 404	11
STM3001	Milbourne et al., 1998	(TC)5	79 - 134	10

STM3009	Milbourne et al., 1998	(TC)13	135 - 159	7
STM3012	Milbourne et al., 1998	(CT)4…(CT)8	166 - 208	6
STM3016	Milbourne et al., 1998	(GA)27	102 - 156	7
STM3020	Milbourne et al., 1998	(CT)10	257	1
STM3023	Milbourne et al., 1998	(GA)9…(GA)8…(GA)4	119 - 195	5
STM5121	Ghislain et al., 2009	(TGT)5	281 - 293	7
STM5127	Ghislain et al., 2009	(TCT)5	200 - 272	11
STM5130	Ghislain et al., 2009	(GCG)5	243 - 261	6
STM5136	Reid and Kerr, 2007	(AGA)5	215 - 247	7
STM5140	Bradshaw et al., 2006	(AAT)7	161 - 194	8
STM5148	Ghislain et al., 2009	(GAA)17	403 - 475	15
STPATP1	Provan et al., 1996	(AT)22	166 - 191	8
STPoAc58	Ghislain et al., 2004	(TA)13	227 - 244	8
STPRINPSG	Provan et al., 1996	(TA)23	143 - 160	5
STSNRNA10	Provan et al., 1996	(A)19	168 - 177	6
STWAX-2	Ghislain et al., 2004	(ACTC)5	209 - 243	12
TC74728	Kørup Sørensen et al., 2007	(CGG)7	71 - 88	7
TC75529	Kørup Sørensen et al., 2007	(GCC)8	74 - 85	6
TC77529	Kørup Sørensen et al., 2007	(GAT)7	122 - 155	7
TC84379	Kørup Sørensen et al., 2007	(GCA)7	86 - 100	4
TC84781	Kørup Sørensen et al., 2007	(GTA)7	210 - 219	4
TC85064	Kørup Sørensen et al., 2007	(TTC)7	143 - 153	4

Table S3. List of the subset of 134 pairwise comparisons where the difference between the individuals is ≤ 5.

**Accession 1**	**Accession 2**	**No. differences**
Æggeblomme LKF	Æggeblomme NGB	0
Asparges NGS	Asparges LKF	0
Beate	Kavlås	0
Blå fra Onsøy	Blå Kerrs Pink	0
Blaar Islenskar	Svart Valdres	0
Congo (SWE)	Congo LKF	0
Congo (SWE)	Svartpotet fra Vegårshei	0
Congo LKF	Svartpotet fra Vegårshei	0
Early Rose NGS	Tranås	0
Eigenheimer	Granuddspotatis	0
Folva	Ledsjö Gul	0
Gamle Raude fra Aurland	Gammelraude	0
Gul Kvæfjording	Rød Kvæfjording	0
Gullauge Gul variant I	Gullauge Gul variant II	0
Gullauge Gul variant I	Gullauge Rød	0
Gullauge Gul variant I	Röd Mandel	0
Gullauge Gul variant II	Gullauge Rød	0
Gullauge Gul variant II	Röd Mandel	0
Gullauge Rød	Röd Mandel	0
Hårek NGB	Hårek NGS	0
Himalaya	I-1039	0
Kerrs Pink med blått skall	Luröpotatis	0
Kerrs Pink med blått skall	Pålle Kättilstorp	0
Luröpotatis	Pålle Kättilstorp	0
Maria	Superb	0
Prestkvern NGB	Prestkvern NGS	0
Purple Peruvian	Vitelotte	0
Reyð Epli	Vallgren Falbygden	0
Ringerikspotet variant I	Ringerikspotet variant II	0
Ringerikspotet variant I	Ringerikspotet variant III	0
Ringerikspotet variant II	Ringerikspotet variant III	0
Russet Burbank NGS	Russet Burbank LKF	0
Foula Red	Gul Kvæfjording	1
Jonsok	Løgumkloster	1
Karjalan Musta	Pålle Kättilstorp	1
Marius II	Marius	1
Pålle Kättilstorp	Reyð Epli	1
Pålle Kättilstorp	Vallgren Falbygden	1
Ameriker	Early Rose NGS	1
Ameriker	Tranås	1
Arran Victory	Kerrs Pink med blått skall	1
Arran Victory	Luröpotatis	1
Blå Kartoffel (DDSF)	Purple Peruvian	1
Blå Kartoffel (DDSF)	Vitelotte	1
Early Rose NGB	Early Rose NGS	1
Early Rose NGB	Tranås	1
Early Rose NGS	Fljota	1

Fakse	Per Larsgården	1
Fljota	Tranås	1
Foula Red	Rød Kvæfjording	1
Jøssing NGB	Jøssing NGS	1
Karjalan Musta	Kerrs Pink med blått skall	1
Karjalan Musta	Luröpotatis	1
Kavlås	Olofstorp	1
Kerrs Pink med blått skall	Reyð Epli	1
Kerrs Pink med blått skall	Vallgren Falbygden	1
Lillhärjåbygget	Mandel variant II (Ekrann)	1
Luröpotatis	Reyð Epli	1
Luröpotatis	Vallgren Falbygden	1
Raudar Islenskar (Gammal Svensk Röd)	Små Röda	1
Reyð Epli	Weinberger Blaue	1
Ringerikspotet variant I	Ringerikspotet NGB	1
Ringerikspotet variant II	Ringerikspotet NGB	1
Ringerikspotet variant III	Ringerikspotet NGB	1
Roslanglänna	Tysk Blå	1
Vallgren Falbygden	Weinberger Blaue	1
Beate	Olofstorp	2
Blå Dalsland	Blå Torpar	2
Gullauge Gul variant I	Gullauge LKF	2
Gullauge Gul variant II	Gullauge LKF	2
Gullauge Rød	Gullauge LKF	2
Gullauge LKF	Röd Mandel	2
Lange's potet	Tidlig Blå fra Halden	2
Ameriker	Early Rose NGB	2
Ameriker	Fljota	2
Arran Victory	Pålle Kättilstorp	2
Blå fra Onsøy	Kerrs Pink med blått skall	2
Blå fra Onsøy	Luröpotatis	2
Blå fra Onsøy	Pålle Kättilstorp	2
Blå fra Onsøy	Weinberger Blaue	2
Blå Kerrs Pink	Kerrs Pink med blått skall	2
Blå Kerrs Pink	Luröpotatis	2
Blå Kerrs Pink	Pålle Kättilstorp	2
Blå Kerrs Pink	Weinberger Blaue	2
Early Puritan	Jonsok	2
Early Rose NGB	Fljota	2
Early Rose NGS	Løgumkloster	2
Karjalan Musta	Reyð Epli	2
Karjalan Musta	Vallgren Falbygden	2
Kerrs Pink med blått skall	Weinberger Blaue	2
King Edward VII	Raude fra Skjåk	2
Løgumkloster	Tranås	2
Luröpotatis	Weinberger Blaue	2
Mandel variant II (Ekrann)	Mandel variant III (klon 6)	2
Pålle Kättilstorp	Weinberger Blaue	2
Ameriker	Løgumkloster	3
Arran Victory	Karjalan Musta	3
Blå fra Onsøy	Karjalan Musta	3
Blå Kerrs Pink	Karjalan Musta	3

Foula Red	Rød Kvæfjord	3
Semlo NGB	Semlo LKF	3
Arran Victory	Blå fra Onsøy	3
Arran Victory	Blå Kerrs Pink	3
Arran Victory	Reyð Epli	3
Arran Victory	Vallgren Falbygden	3
Arran Victory	Weinberger Blaue	3
Åspotet NGB	Åspotet NGS	3
Blå fra Onsøy	Reyð Epli	3
Blå fra Onsøy	Vallgren Falbygden	3
Blå Kerrs Pink	Reyð Epli	3
Blå Kerrs Pink	Vallgren Falbygden	3
Early Puritan	Early Rose NGS	3
Early Puritan	Løgumkloster	3
Early Puritan	Tranås	3
Early Rose NGS	Jonsok	3
Jonsok	Tranås	3
Karjalan Musta	Weinberger Blaue	3
Mandel variant I (klon 1)	Mandel variant II (Ekrann)	3
Early Rose NGB	Løgumkloster	3
Fljota	Løgumkloster	3
Mandel variant I (klon1)	Mandel variant III (klon 6)	3
Early Puritan	Fljota	4
Ameriker	Early Puritan	4
Ameriker	Jonsok	4
Early Puritan	Early Rose NGB	4
Early Rose NGB	Jonsok	4
Fljota	Jonsok	4
Gul Kvæfjording	Rød Kvæfjord	4
King Edward VII	Norska Röda	4
Lillhärjåbygget	Mandel variant III (klon 6)	4
Norska Röda	Raude fra Skjåk	4
Rød Kvæfjord	Rød Kvæfjording	4
Kiva	Tylva	5
Lillhärjåbygget	Mandel variant I (klon1)	5
