# Supplementary material for: Genetic markers identify duplicates in Nordic potato collections
Source: Front Plant Sci. 2024 Aug 26;15:1405314. doi: 10.3389/fpls.2024.1405314 (PMC11381411; doi:10.3389/fpls.2024.1405314)

***Supplementary Figure S1.*** Dendrogram constructed with the Neighbour joining method with branches with less than 75% bootstrap support collapsed

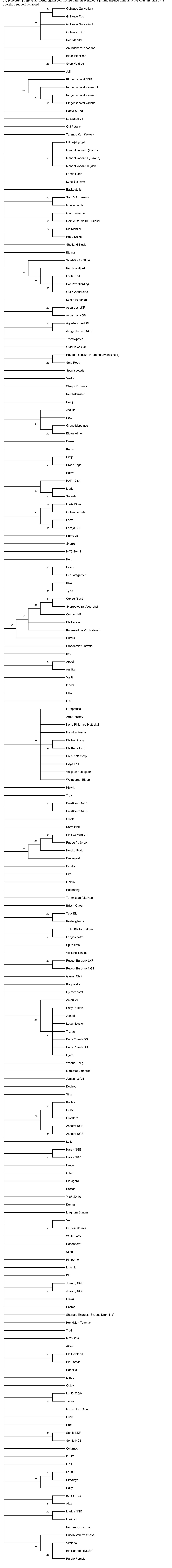

Supplement: Supplementary file 2 [file DataSheet2.pdf]
